# Relationship Between MCP‐1 Levels in GCF and Periodontitis: A Systematic Review With Meta‐Analysis and Analysis of Molecular Interactions

**DOI:** 10.1111/jcmm.70545

**Published:** 2025-05-08

**Authors:** Mario Alberto Alarcón‐Sánchez, Ruth Rodríguez‐Montaño, Sarah Monserrat Lomelí‐Martínez, Artak Heboyan

**Affiliations:** ^1^ Molecular Biology Department, University Center of Health Sciences University of Guadalajara (CUCS‐UdeG) Guadalajara Jalisco Mexico; ^2^ Institute of Research in Dentistry, Department of Integral Dental Clinics University Center of Health Sciences, University of Guadalajara (CUCS‐UdeG) Guadalajara Jalisco Mexico; ^3^ Department of Health and Illness as an Individual and Collective Process, University Center of Tlajomulco University of Guadalajara (CUTLAJO‐UdeG) Tlajomulco de Zuñiga Jalisco Mexico; ^4^ Department of Medical and Life Sciences, La Ciénega University Center University of Guadalajara Ocotlán Jalisco Mexico; ^5^ Department of Prosthodontics, Faculty of Stomatology Yerevan State Medical University after Mkhitar Heratsi Yerevan Armenia; ^6^ Department of Research Analytics, Saveetha Dental College and Hospitals, Saveetha Institute of Medical and Technical Sciences Saveetha University Chennai India; ^7^ Department of Prosthodontics, School of Dentistry Tehran University of Medical Sciences Tehran Iran

**Keywords:** biomarkers, gingival crevicular fluid, monocyte chemoattractant protein 1, periodontal disease, periodontitis

## Abstract

Monocyte chemoattractant protein 1 (MCP‐1) is involved in monocyte chemotaxis, endothelial activation and regulation of leukocyte function in biological activities that promote inflammation, such as in periodontitis. A systematic review and meta‐analysis was conducted with the primary objective of investigating the roles of MCP‐1 in the gingival crevicular fluid (GCF) of subjects with chronic periodontitis compared to periodontally healthy subjects. The study protocol adhered to PRISMA guidelines. Digital searches were carried out across several databases, including PubMed, Dentistry & Oral Science Source, ScienceDirect, Scopus, Web of Science and Google Scholar. The quality of the studies was evaluated using the JBI tool for cross‐sectional studies and clinical trials. To assess the concentration of MCP‐1 in the GCF of the exposure group versus the control group, a meta‐analysis was conducted employing a random‐effects model. The search strategy yielded 1694 articles, with 14 studies meeting the inclusion criteria and 10 articles subjected to quantitative analysis. A total of 497 subjects were examined, comprising 298 cases and 199 controls. The meta‐analysis indicated a significant increase in MCP‐1 levels in the GCF of individuals with chronic periodontitis compared to healthy subjects (GCF: SMD = 20.29, 95% CI: 10.33–30.25, *Z* = 3.992, *p* = 0.001*). GCF MCP‐1 levels are elevated in periodontitis compared to healthy controls, suggesting its potential future use as a diagnostic tool in clinical settings.

**Abbreviations**
CCL20: C‐C motif chemokine 20
CCL5: C‐C motif chemokine 5
CCR1: C‐C chemokine receptor type 1
CCR2: C‐C chemokine receptor type 2
CCR5: C‐C chemokine receptor type 5
CSF2: granulocyte‐macrophage colony‐stimulating factor
CX3CR1: CX3C chemokine receptor 1
CXCL1: C‐X‐C motif chemokine 1
CXCL10: C‐X‐C motif chemokine 10
CXCL4: C‐X‐C motif chemokine 4
CXCL8: C‐X‐C motif chemokine 9
CXCR2: C‐X‐C chemokine receptor type 2
CXCR4: C‐X‐C chemokine receptor type 4
ICAM‐1: intercellular adhesion molecule 1
IFN‐γ: interferon gamma
IL‐10: interleukin 10
IL‐1α: interleukin 1 alpha
IL‐1β: interleukin 1 beta
IL‐4: interleukin 4
IL‐6: interleukin 6
TNF‐α: tumour necrosis factor alpha
VCAM‐1: vascular cell adhesion protein 1

## 1 Introduction

Periodontitis is a chronic, infectious and inflammatory disease impacting the teeth's supporting structures, known as the periodontium [1]. It manifests through symptoms, such as gingival inflammation, the formation of periodontal pockets, loss of attachment, bleeding, suppuration upon probing or spontaneously, tooth mobility and various patterns of bone loss [2]. Affecting a significant portion of the population (64.3%), it considerably influences the oral health‐related quality of life, as well as the psychosocial and economic conditions of those susceptible [3, 4, 5]. The disease is caused by obligate gram‐negative anaerobes ( *Fusobacterium nucleatum* , *Prevotella intermedia* , *Aggregatibacter actinomycetemcomitans* , *Tannerella forsythia* , *Treponema denticola* and *Porphyromonas gingivalis* ), which release substances that activate the host's innate and adaptive immune systems. This activation leads to the production of various molecules like enzymes, cytokines and chemokines, intensifying the immunoinflammatory and degenerative processes in the periodontium [6, 7].

**FIGURE 1:**
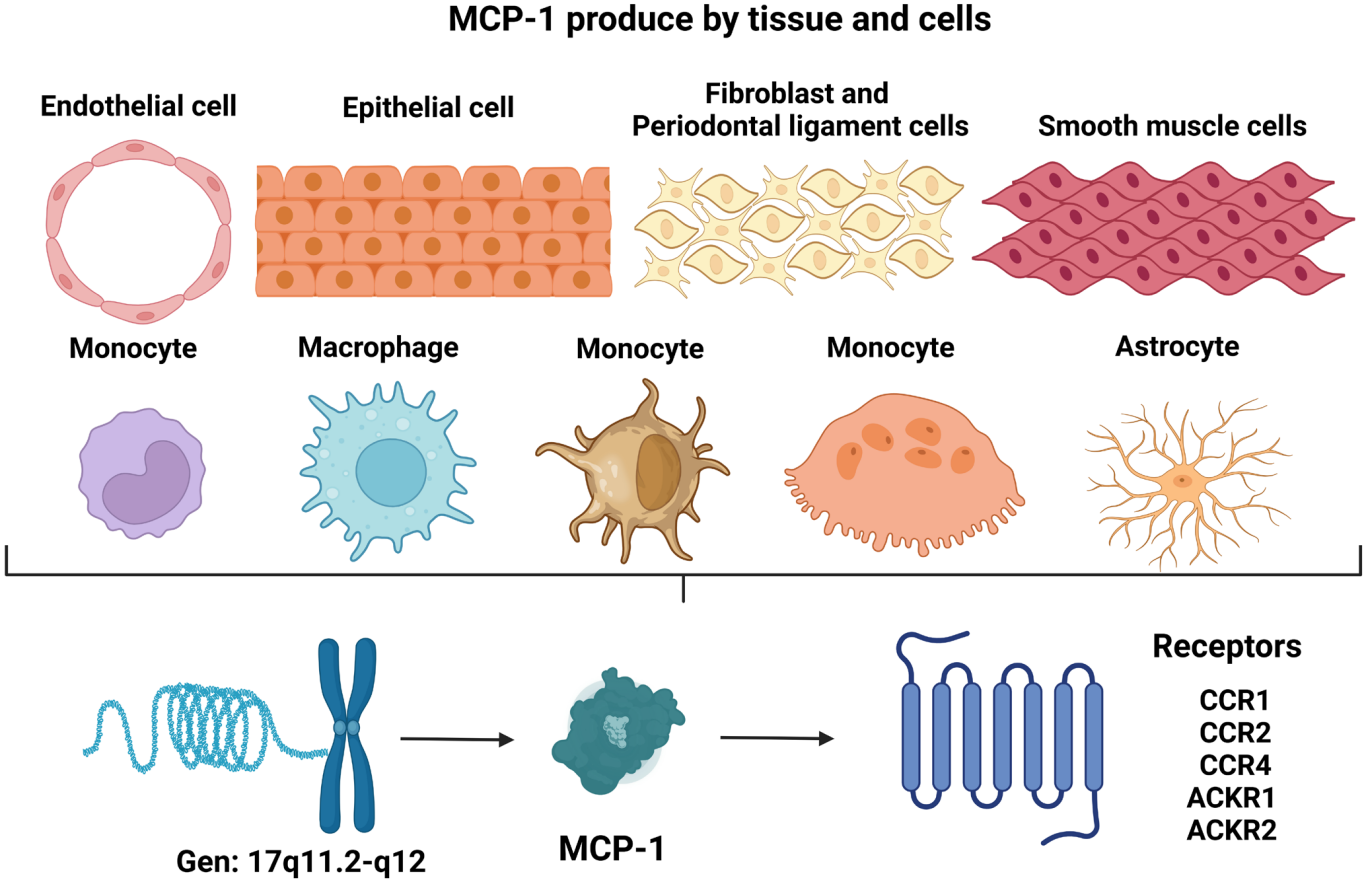
MCP‐1 cells production and receptors. MCP‐1/CCL2 is a chemokine that can be expressed by a wide variety of cells such as various tissues, such as cells of the immune system. CCL2 can bind to a variety of receptors, including classical chemokine receptors such as CCR1, CCR2, CCR4 and atypical chemokine receptors ACKR1, ACKR2.

**FIGURE 2:**
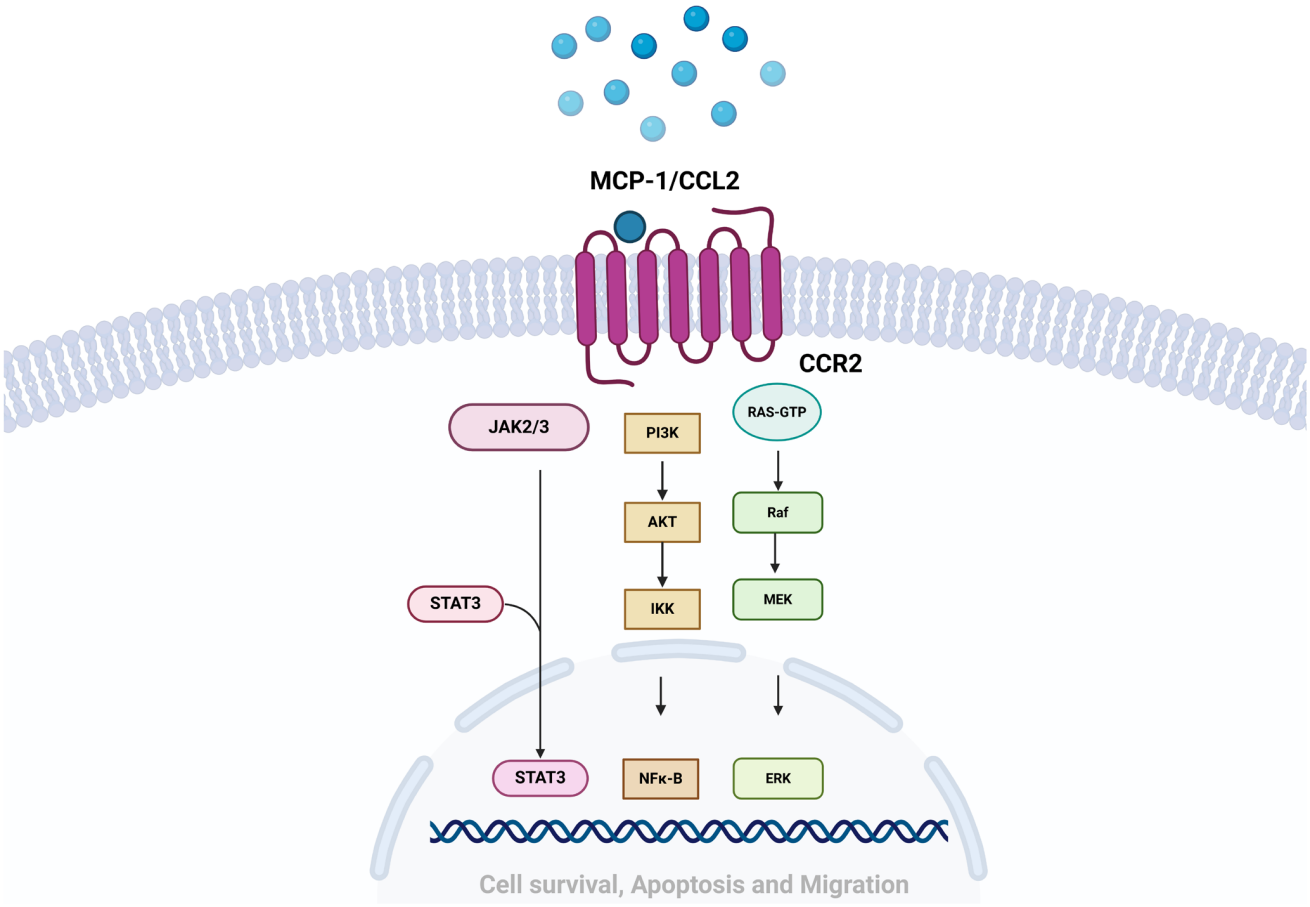
MCP‐1 signalling. Several signalling pathways, including the JAK/STAT, MAPK and PI3K/Akt pathways, are activated when CCR2 binds to its ligand, MCP‐1. Numerous transcription factors and genes involved in cytokine production, growth, cell differentiation, cell survival, migration and apoptosis are activated as a result.

Chemokines, a family of small proteins with molecular weights ranging from 8 to 14 kDa, are secreted by the immune system and primarily regulate cell movement in response to chemical stimuli [8]. They are categorised into four families: two major subgroups (CXC and CC) and two minor subgroups (CX3C and C), based on the cysteine residues at the N‐terminal end [9]. Monocyte chemoattractant protein 1 (MCP‐1), also known as C‐C motif chemokine ligand 2 (CCL2), is a chemokine crucial for monocyte chemotaxis, endothelial activation and leukocyte function regulation, which are key in inflammation and periodontitis [10]. The *MCP1* gene, encoding this protein, is located at 17q11.2‐q12 [11]. MCP‐1 precursor is a hydrophobic amino‐terminal signal peptide comprising 23 amino acids, while the mature protein has 76 amino acids and a molecular weight of 11.02 kDa [12]. Produced mainly by epithelial cells, endothelial cells, smooth muscle cells, astrocytes, monocytes/macrophages, dendritic cells, gingival fibroblasts, periodontal ligament cells and osteoclasts [13, 14, 15], MCP‐1, upon release, binds with the C‐C chemokine receptor type 2 (CCR2). This interaction activates various signalling pathways, including the nuclear factor kappa B (NF‐κB), phosphatidylinositol 3‐kinase (PI3K/Akt) and extracellular signal‐regulated kinase (ERK) pathways. These pathways facilitate the migration of inflammatory cells to sites of infection, inflammation and tissue destruction within periodontal tissues, as depicted in Figures 1 and 2 [11, 12].

Diagnosis of periodontitis is performed by a series of clinical tests, such as periodontal probing and radiographic evaluation; however, these tools alone do not discriminate between disease onset and progression. Therefore, considering the constantly increasing number of subjects with periodontitis, investigators are looking for new molecular tools to diagnose, assess disease severity, treatment efficacy and monitor the risk of recurrence [16].

Gingival crevicular fluid (GCF) has great potential as a diagnostic biofluid and offers some advantages over saliva, gingival tissue biopsies, serum and plasma due to its easy collection, which is non‐invasive and accessible in the gingival sulcus, also by the preparation of aliquots with a sufficient amount of sample for the determination of different inflammatory mediators, high sensitivity and specificity, cost‐effectiveness, storage and early transport [17, 18, 19]. Biomarkers in GCF are important for determining the presence, risk and progression of periodontitis [20]. The clinical application of biomarker technology from research settings to clinical practice necessitates studies that pinpoint biomarkers linked to both healthy and diseased periodontal states [21]. Research has indicated elevated levels of tumour necrosis factor alpha (TNF‐α), interleukin 1 beta (IL‐1β), interleukin 6 (IL‐6), interleukin 8 (IL‐8), interleukin 17 (IL‐17), interleukin 18 (IL‐18), interleukin 23 (IL‐23), as well as fractalkine (CX3CL1) and its receptor (CX3CR1) in the GCF of individuals with periodontitis compared to those with a healthy periodontium [22, 23, 24, 25, 26, 27]. Additionally, given that MCP‐1 expression typically rises in immunoinflammatory conditions, a multitude of studies have documented fluctuations in MCP‐1 levels in the GCF of individuals with and without periodontitis [28, 29, 30, 31, 32, 33, 34, 35, 36, 37, 38, 39, 40, 41]. Yet, as of now, there is an absence of a comprehensive systematic review that includes a meta‐analysis to consolidate the results of these studies. Therefore, based on the previously described background, we set out the following three objectives:

- To compile all available scientific evidence through a systematic review on the role of MCP‐1 in the pathogenesis of periodontitis.
- To compare the levels of MCP‐1 in GCF of subjects with periodontitis and healthy controls by means of a meta‐analysis.
- To perform a bioinformatics analysis using the STRING database to evaluate MCP‐1 interaction networks in biological processes related to periodontal disease and other oral conditions.

## 2 Methodology

### 2.1 Protocol and Permission

The present review was prepared following the Preferred Reporting Items for Systematic Reviews and Meta‐Analysis (PRISMA) guidelines [42]. The protocol was registered in the Open Science Framework (OSF) [43] with registration number: https://doi.org/10.17605/OSF.IO/UC72F.

### 2.2 PECO Items and Research Question

The research question was constructed using the PECO items (Population, Exposure, Comparison and Outcomes):

- (P): Systemically healthy subjects, with and without chronic periodontitis.
- (E): Systemically healthy subjects with chronic periodontitis.
- (C): Systemically healthy subjects without chronic periodontitis.
- (O): MCP‐1 levels in GCF.

The question was as follows: Are there changes in MCP‐1 levels in GCF of subjects with chronic periodontitis compared to periodontally healthy subjects?

### 2.3 Elegibility Criteria

Inclusion criteria were cross‐sectional and randomised clinical trials published in the English language, reporting MCP‐1 levels in GCF of periodontitis subjects with a probing depth (PD) ≥ 4 mm and a clinical attachment level (CAL) ≥ 3 mm. Studies published before 1990 were excluded, as well as studies analysing MCP‐1 levels in GCF or any other biological sample (saliva, gingival tissue, plasma and serum) from subjects with gingivitis, aggressive periodontitis and peri‐implantitis. Systemically compromised subjects, smokers, pregnant women, subjects undergoing orthodontic treatment, as well as those participants taking antibiotics or immunomodulators were also excluded. Systematic reviews, meta‐analyses, comprehensive, narrative and scoping reviews, as well as editorials, short communications, conference papers, case reports and/or case series were also excluded from the present study.

### 2.4 Search Strategy and Study Selection

An exhaustive literature search was conducted in six databases, including PubMed, Dentistry & Oral Science Source, ScienceDirect, Scopus, Web of Science and Google Schoolar from November 15th, 1993 to September 10th, 2021. The search strategy used for PubMed was as follows: (((‘Chemokine CCL2’[Mesh]) AND ‘Gingival Crevicular Fluid’[Mesh])) AND ‘Periodontal Diseases’[Mesh]. For the rest, the keywords used were: ‘MCP‐1’, ‘Gingival Crevicular Fluid’ and ‘Periodontitis’. In addition, a secondary hand search was performed in the following Journals: *Periodontology 2000*, *Journal of Clinical Periodontology*, *Journal of Periodontology*, *Journal of Periodontal Research*, *Journal of Periodontal and Implant Science* and *International Journal of Periodontics & Restorative Dentistry*.

The articles resulting from the above process were reviewed by examining titles, abstracts and full text.

### 2.5 Data Extraction

Two investigators (R.R.M and S.M.L.M) independently extracted the data. The obtained information was as follows: First author's data, year of publication, country, type of study, approval by the ethics committee of the corresponding institution, journal name, inclusion and exclusion criteria, gender and age of participants, as well as number of cases (subjects with chronic periodontitis) and number of controls (periodontally healthy subjects). Information was also collected on the type of classification used for the diagnosis of periodontitis, the clinical parameters evaluated, the type of GCF sampling, the immunoassay technique for the determination of MCP‐1 levels, the mean values (pg/mL) of MCP‐1 in both the exposure and control groups, the statistical significance and the main results obtained. All data were tabulated in tables constructed using Excel software (Microsoft).

### 2.6 Study Quality Assessment

The quality of the studies was evaluated independently by two investigators (M.A.A.S and R.R.M). The Joanna Briggs Institute (JBI) tool was used to assess cross‐sectional studies and randomised clinical trials [44]. For the first segment of studies, eight questions were evaluated, whereas for the second segment, 13 were evaluated. In both, the final percentage was obtained, and if the result was between 0% and 49%, there was a low quality assessment; if it was between 50% and 69%, there was a moderate quality assessment; and if the result was ≥ 70%, there was a high quality assessment. These assessments were confirmed by a third researcher (A.H).

### 2.7 Statistical Analysis

For statistical analysis, STATA V.18 software (StataCorp, College Station, TX, USA) was employed. A *p* value of less than 0.05* was deemed statistically significant. The standardised mean difference (SMD) with 95% confidence intervals (CI) was calculated using a random/fixed effects model, depending on the heterogeneity value (> 50% indicating high heterogeneity), estimated by the *Q* statistic and quantified with the *I* ^2^ statistic. Forest plots were used to illustrate the estimates with 95% CI, and publication bias was evaluated using funnel plots and Egger linear regression.

### 2.8 Prediction of Protein Interaction Networks

An interaction network analysis using STRING provided insights into the relationships between MCP‐1 and other proteins, as well as its links to periodontal disease and various oral conditions. STRING is a database that catalogues both known and predicted protein–protein interactions. These interactions may be direct, as in physical connections, or indirect, indicating functional associations. They are derived from computational predictions, knowledge transfer between organisms, and a compilation of primary database interactions [45].

## 3 Results

**FIGURE 3:**
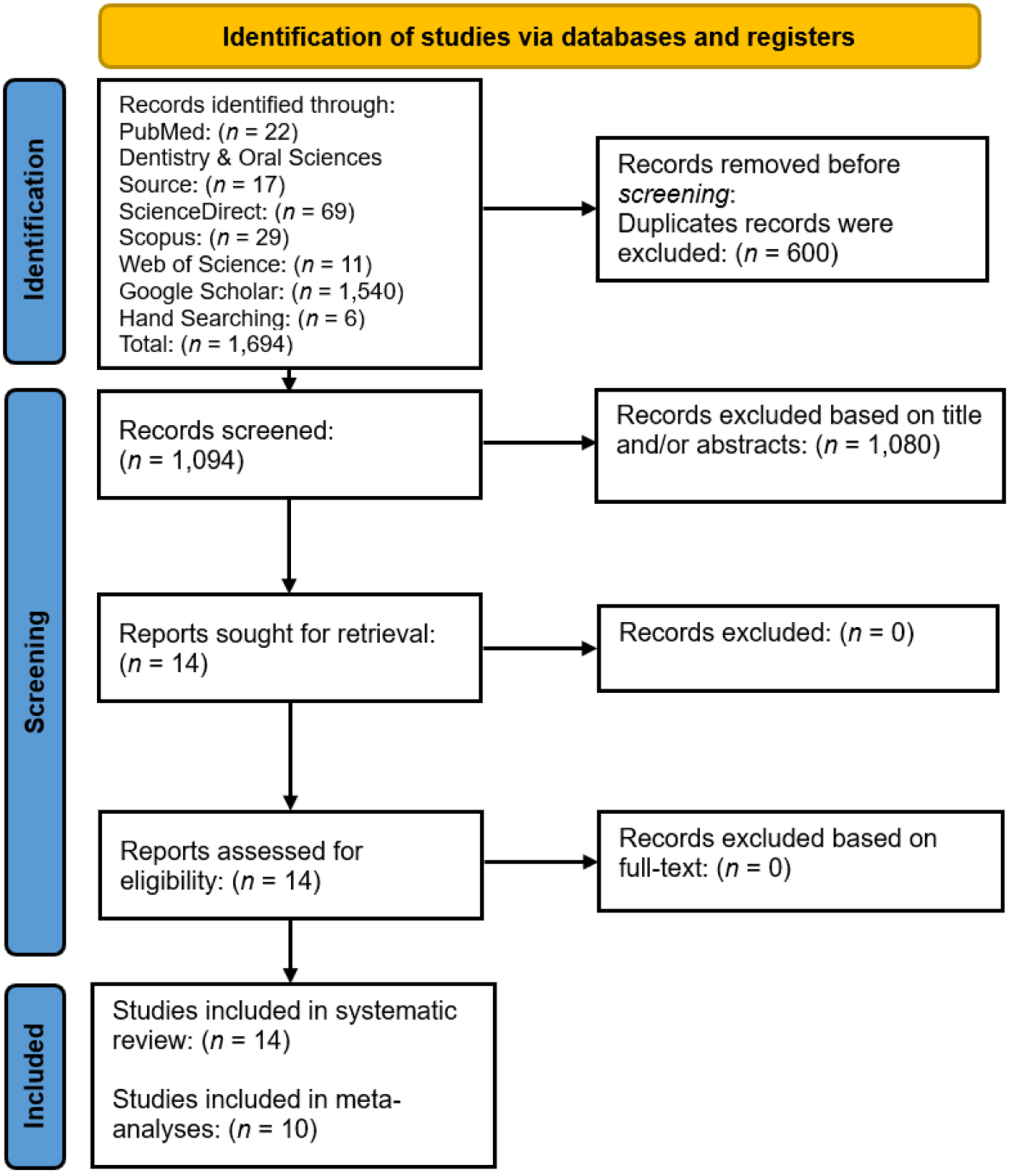
PRISMA 2020 flow diagram for study selection. MCP‐1/CCL‐2 interactome.

Initially, 1694 articles were identified across six databases: PubMed (22 articles), Dentistry & Oral Sciences Source (17 articles), Science Direct (69 articles), Scopus (29 articles), Web of Science (11 articles), Google Scholar (1540 articles) and through hand searching (6 articles). After removing duplicates (600 articles) and screening by title and abstract (1080 articles), 14 remaining full‐text studies were examined. Ultimately, eight articles were considered eligible, supplemented by 6 articles from hand searching, totaling 14 articles for qualitative synthesis and 10 for quantitative analysis in this review. The study selection process is depicted in Figure 3.

This study reviewed 11 cross‐sectional studies [28, 29, 30, 31, 32, 33, 36, 37, 40, 41] and three randomised clinical trials [34, 35, 38]. A total of 497 subjects were included in the reviewed research, with 298 in the exposure group (individuals with chronic periodontitis) and 199 in the control group. The subjects' ages ranged from 20 to 75 years, with an average age of 40.83 ± 5.80 years. Among them, 39.2% were male, 31.4% were female, and for 29.4%, the gender was not specified [29, 32, 33, 35, 41]. The majority of the studies (10 out of 14, or 71.42%) were published post‐2009 [28, 29, 30, 31, 32, 33, 34, 35, 36, 37]. The earliest study dated back to 1993 [41], while the latest was from 2021 [28]. All studies received approval from their respective institutions' ethics committees [28, 29, 30, 31, 32, 33, 34, 35, 36, 37, 38, 39, 40, 41]. The most common exclusion criteria were the presence of systemic diseases and the use of antibiotics or immunomodulators (78.57%), which could influence periodontal health [28, 29, 32, 34, 35, 36, 37, 38, 39, 41]. The 14 articles originated from seven different countries, with four studies from India [32, 34, 36, 37], three from Japan [33, 40, 41], two each from Turkey [28, 39] and Saudi Arabia [29, 30] and one each from China [31], the USA [35] and Chile [38]. The publication journals are listed in Table 1.

**TABLE 1:** Clinical and demographic characteristics of included studies.

Author/year	Country	Study type	E‐A	Journal type	Inclusion criteria	Exclusion criteria	Gender F^e^/M^a^	Age (M/R)	*n* (CG/EG)	*n* (Total)
Gündoĝar et al. 2021 [28]	Turkey	Cross‐sectional	Yes	*Cent. Eur. J. Immunol*.	Periodontitis subjects	Systemic disorders, smokers, pregnant, antibiotics and immunomodulatory drugs and history of periodontal treatment	26/23	20–58	25/24	49
Patil et al. 2021 [29]	Saudi Arabia	Cross‐sectional	Yes	*Int. J. Mol. Sci*.	Periodontitis subjects	Systemic disorders, smokers, pregnant, antibiotics and immunomodulatory drugs and history of periodontal treatment	NR	NR	5/5	10
Fageeh et al. 2021 [30]	Saudi Arabia	Cross‐sectional	Yes	*Saudi J. Biol. Sci*.	Periodontitis subjects	NR	4/6	36–52	5/5	10
Zhu et al. 2015 [31]	China	Cross‐sectional	Yes	*Int. J. Clin. Exp. Pathol*.	Periodontitis subjects	Systemic disorders, smokers and drinking alcohol	30/25	43.15	28/27	55
Anil et al. 2013 [32]	India	Cross‐sectional	Yes	*J. Periodontol*.	Periodontitis subjects	Systemic disorders, antibiotics and immunomodulatory drugs	NR	25–55	30/30	60
Shimada et al. 2013 [33]	Japan	Cross‐sectional	Yes	*Arch. Oral Biol*.	Periodontitis subjects	Antibiotics and immunomodulatory drugs and history of periodontal treatment	NR	NR	11/22	23
Gupta et al. 2013 [34]	India	Clinical trial	Yes	*Cytokines*	Periodontitis subjects	Systemic disorders, smokers, pregnant, antibiotics and immunomodulatory drugs and history of periodontal treatment	21/24	42.56	15/30	45
Thunell et al. 2010 [35]	USA	Clinical trial	Yes	*J. Periodontal. Res*.	Periodontitis subjects	Systemic disorders, smokers, pregnant, antibiotics and immunomodulatory drugs and history of periodontal treatment	NR	40–75	6/6	12
Pradeep et al. 2009 [36]	India	Cross‐sectional	Yes	*Arch. Oral Biol*.	Periodontitis subjects	Systemic disorders, smokers, antibiotics and immunomodulatory drugs and history of periodontal treatment	20/20	28–42	20/20	40
Pradeep et al. 2009 [37]	India	Cross‐sectional	Yes	*J. Periodontol*.	Periodontitis subjects	Systemic disorders, smokers, antibiotics and immunomodulatory drugs and history of periodontal treatment	20/20	32.8	20/20	40
Silva et al. 2008 [38]	Chile	Clinical trial	Yes	*J. Clin. Periodontol*.	Periodontitis subjects	Systemic disorders, antibiotics and immunomodulatory drugs and history of periodontal treatment	14/5	45.66	18/18	36
Kurtis et al. 2005 [39]	Turkey	Cross‐sectional	Yes	*J. Periodontol*.	Periodontitis subjects	Systemic disorders, smokers, antibiotics and immunomodulatory drugs	21/24	34.42	20/25	45
Hanioka et al. 2000 [40]	Japan	Cross‐sectional	Yes	*J. Clin. Periodontol*.	Periodontitis subjects	Systemic disorders	0/48	46.4	0/48	48
Hanazawa et al. 1993 [41]	Japan	Cross‐sectional	Yes	*Infect Immun*.	Periodontitis subjects	Antibiotics and immunomodulatory drugs and history of periodontal treatment	NR	NR	6/18	24

Abbreviations: CG, control group; E‐A, ethical approval; EG, exposure group; F^e^, female; *M*, mean; M^a^, male; NR, not reported; R, range.

The eighty‐five point 71% of the studies do not mention the type of classification used to determine the periodontal condition [30, 31, 32, 33, 34, 35, 36, 37, 38, 39, 40, 41]. The most frequently evaluated clinical parameters were PD (71.42%) [28, 31, 32, 33, 34, 35, 36, 37, 39, 40], followed by CAL (64.28%) [28, 31, 32, 33, 34, 35, 36, 37, 39]. Regarding the way of collecting GCF samples, 70% of the studies used paper strips [28, 31, 33, 35, 38, 39, 40], while 42.85% used microcapillary pipettes [29, 32, 34, 36, 37, 41]. Regarding the immunoassay technique used, 64.28% of the studies employed the ELISA technique [30, 31, 32, 34, 36, 37, 38, 39, 40], followed by the multiplex based on fluorescent microspheres (21.42%) [28, 33, 35]. Overall, qualitative analysis of all studies demonstrated increased levels of MCP‐1 in GCF of subjects with chronic periodontitis compared to periodontally healthy subjects [28, 29, 30, 31, 32, 33, 34, 35, 36, 37, 38, 39, 40, 41] (Table 2).

**TABLE 2:** Periodontal profile and main features of monocyte chemoattractant protein‐1 (MCP‐1) in gingival crevicular fluid of chronic periodontitis and periodontally healthy subjects.

Study/year	Classification	Periodontal criteria (CP)	Clinical parameters assessment	GCF sampling	Methods	Value CG pg/mL	Value CP pg/mL	*p*	Main results
Gündoĝar et al. 2021 [28]	1999 AAP	NR	GI, PI, PD, CAL	Paper strips	Bio‐Plex multiplexing	5.62 (3.91)	10.64 (4.39)	< 0.05	↑ MCP‐1 levels in group with EG compared to CG
Patil et al. 2021 [29]	2018 World Workshop	NR	NR	Microcapillary pipettes	Cytometric bead array	4.88 (1.04)	16.29 (2.92)	< 0.01	↑ MCP‐1 levels in group with EG compared to CG
Fageeh et al. 2021 [30]	NR	NR	NR	NR	ELISA (biosystems)	NR	NR	< 0.05	↑ MCP‐1 levels in group with EG compared to CG
Zhu et al. 2015 [31]	NR	PD ≥ 4 mm, BOP	PD, CAL, BOP	Paper strips	ELISA (BioSource)	8.74 (1.47)	16.07 (5.45)	< 0.05	↑ MCP‐1 levels in group with EG compared to CG
Anil et al. 2013 [32]	NR	NR	PD, CAL	Microcapillary pipettes	ELISA (R&D Systems)	19.97 (2.93)	96.43 (5.77)	< 0.05	↑ MCP‐1 levels in group with EG compared to CG
Shimada et al. 2013 [33]	NR	PD 5‐6 mm	PD, CAL, PI, BOP	Paper strips	Multiplex fluorescent bead‐based	1.33 (0.31)	2.53 (0.29)	< 0.01	↑ MCP‐1 levels in group with EG compared to CG
Gupta et al. 2013 [34]	NR	PD ≥ 5 mm, CAL ≥ 6 mm	GI, PI, PD, CAL	Microcapillary pipettes	ELISA (Biotech)	NR	NR	< 0.05	↑ MCP‐1 levels in group with EG compared to CG
Thunell et al. 2010 [35]	NR	CAL ≥ 5 mm	PD, CAL, BOP	Paper strips	Multiplex fluorescent bead‐based	22 (15)	25 (12)	> 0.05	↑ MCP‐1 levels in group with EG compared to CG
Pradeep et al. 2009 [36]	NR	GI > 1, PD > 5 mm	GI, PD, CAL	Microcapillary pipettes	ELISA (R&D Systems)	19.70 (3.27)	72.60 (17.93)	< 0.05	↑ MCP‐1 levels in group with EG compared to CG
Pradeep et al. 2009 [37]	NR	GI > 1, PD ≥ 5 mm, CAL ≥ 3 mm	GI, PD, CAL	Microcapillary pipettes	ELISA (R&D Systems)	20.30 (3.23)	73.30 (17.82)	< 0.05	↑ MCP‐1 levels in group with EG compared to CG
Silva et al. 2008 [38]	NR	PD ≥ 5 mm, CAL ≥ 3 mm	NR	Paper strips	ELISA (ALPCO)	106.12 (19.6)	122.15 (18.4)	> 0.05	↑ MCP‐1 levels in group with EG compared to CG
Kurtis et al. 2005 [39]	NR	NR	PI, GI, PD, CAL	Paper strips	ELISA (CYTELISA)	2.18	4.73	< 0.05	↑ MCP‐1 levels in group with EG compared to CG
Hanioka et al. 2000 [40]	NR	PD ≥ 3 mm	GI, PI, PD	Paper strips	ELISA (Biotech)	—	24.0	—	↑ MCP‐1 levels in group with EG compared to CG
Hanazawa et al. 1993 [41]	NR	CAL ≥ 3 mm	NR	Microcapillary pipettes	Anti‐JE/MCP‐1 antiserum	NR	NR	< 0.01	↑ MCP‐1 levels in group with EG compared to CG

*Note:* The data were reported with mean ± standard deviation.

Abbreviations: AAP, American Academy of Periodontology; CG, control group; EG, exposure group; ELISA, Enzyme‐Linked ImmunoSorbent Assay; MCP‐1, monocyte chemoattractant protein‐1; NR, not reported.

### 3.1 Quality Assessment

The JBI checklist was utilised to evaluate the quality of cross‐sectional and randomised clinical trials. Based on the defined criteria, nine studies (64.28%) exhibited a high quality [28, 31, 32, 33, 36, 37, 39, 40, 41], four studies (28.57%) showed a moderate quality [29, 34, 35, 38] and only one study (7.14%) had a low quality [30] (Tables 3 and 4).

**TABLE 3:** Quality assessment according to the JBI for clinical cross‐sectional studies.

Questions→	1	2	3	4	5	6	7	8	Quality‐Score
Author (year)									
Gündoĝar et al. 2021 [28]	Y	U	Y	U	Y	Y	Y	Y	75%
Patil et al. 2021 [29]	Y	U	Y	U	U	U	Y	Y	50%
Fageeh et al. 2021 [30]	U	U	Y	U	U	U	Y	Y	37.5%
Zhu et al. 2015 [31]	Y	Y	Y	Y	Y	Y	Y	Y	100%
Anil et al. 2013 [32]	Y	U	Y	U	Y	Y	Y	Y	75%
Shimada et al. 2013 [33]	Y	Y	Y	Y	Y	Y	Y	Y	100%
Pradeep et al. 2009 [36]	Y	Y	Y	Y	Y	Y	Y	Y	100%
Pradeep et al. 2009 [37]	Y	Y	Y	Y	Y	Y	Y	Y	100%
Kurtis et al. 2005 [39]	Y	U	Y	U	Y	Y	Y	Y	75%
Hanioka et al. 2000 [40]	Y	Y	Y	Y	U	U	Y	Y	75%
Hanazawa et al. 1993 [41]	Y	Y	Y	Y	U	U	Y	Y	75%

*Note:* Question (Q); N/A, not aplicable; Y, yes; U, unclear. (1) Were the criteria for inclusion in the sample clearly defined? (2) Were the study subjects and the setting described in detail? (3) Was the exposure measured in a valid and reliable way? (4) Were objective, standard criteria used for measurement of the condition? (5) Was confounding factors identified? (6) Were strategies to ideal with confounding factors stated? (7) Were the outcomes measured in a valid and reliable way? (8) Was appropriate statistical analysis used?

**TABLE 4:** Quality assessment according to the JBI for randomised clinical trials.

Questions →	1	2	3	4	5	6	7	8	9	10	11	12	13	Quality‐Score
Author (year)														
Gupta et al. 2013 [34]	N	N	Y	Y	U	U	Y	Y	Y	Y	Y	Y	Y	69.2%
Thunell et al. 2010 [35]	N	N	Y	Y	U	U	Y	Y	Y	Y	Y	Y	Y	69.2%
Silva et al. 2008 [38]	N	N	Y	Y	U	U	Y	Y	Y	Y	Y	Y	Y	69.2%

*Note:* Question (Q); N/A, not aplicable; Y, yes; U, unclear. (1) Was true randomisation used for assigment of participants to treatment groups? (2) Was allocation to treatment groups concealed? (3) Were treatment groups similar at the baseline? (4) Were participants blind to treatment assignment? (5) Were those delivering treatment blind to treatment assigment? (6) Were outcomes assessors blind to treatment assignment? (7) Were treatment groups treated identically other than the intervention of interest? (8) Was follow up complete and if not, were differences between groups in terms of their follow up adequately described and analysed? (9) Were participants analysed in the groups to wich they were randomised? (10) Were outcomes measured in the same way for the treatment groups? (11) Were outcomes measured in a realible way? (12) Was appropriate statistical analysis used? (13) Was the trial design appropiate, and any deviations from the standard RCT design (individual randomisation, parallel groups) accounted for in the conduct and analysis of the trial?

### 3.2 Meta‐Analysis

**FIGURE 4:**
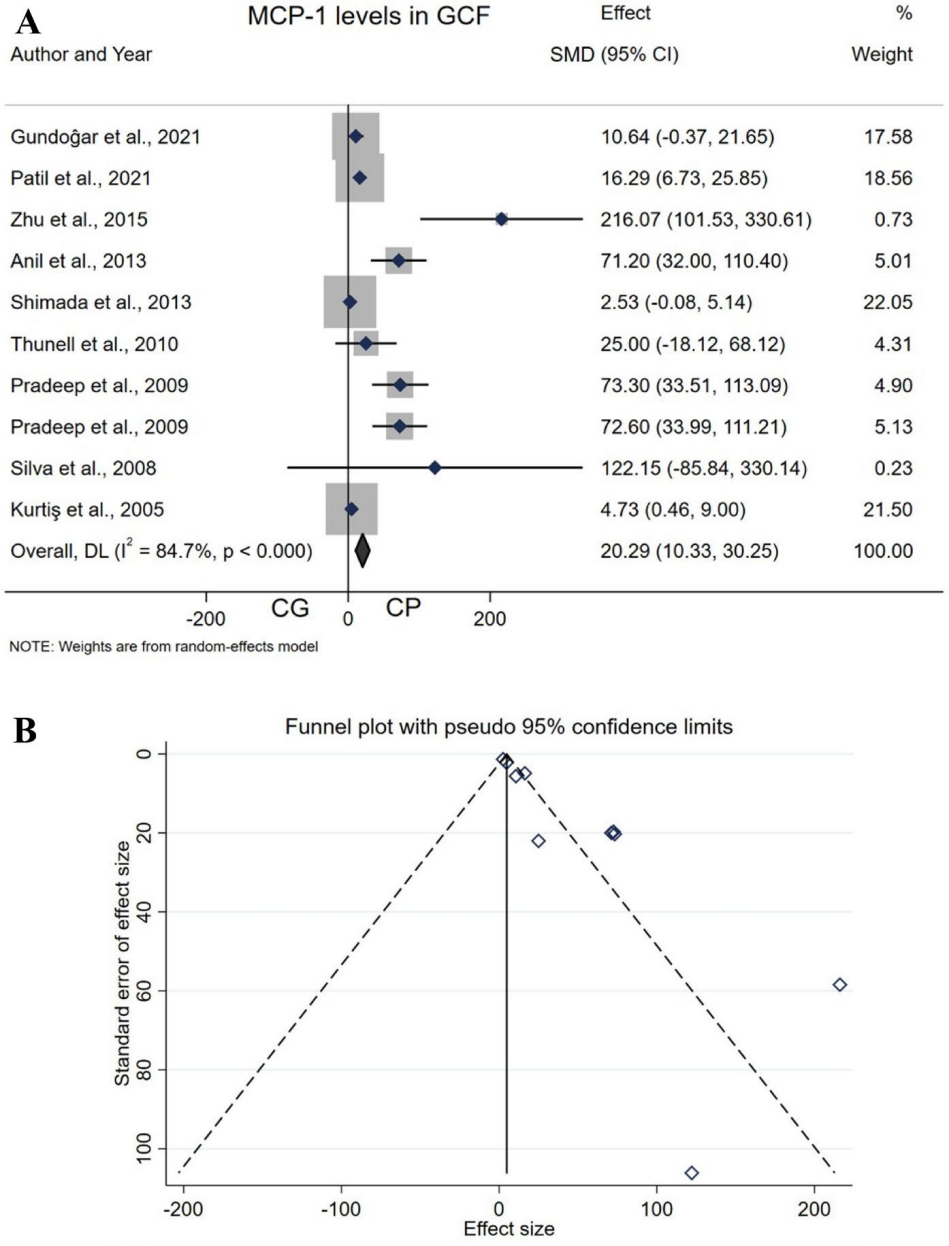
Forest plot comparing GCF MCP‐1 levels of (A) Control group vs. chronic periodontitis. (B) Funnel plot to check the publication bias.

Ten studies [28, 29, 31, 32, 33, 34, 35, 36, 37, 38, 39] compared MCP‐1 levels in GCF between the experimental group (EG, *n* = 197) and control group (CG, *n* = 183). The meta‐analysis results showed a SMD of 20.29 (10.33–30.25), with a *p* < 0.001*, indicating a significant increase in this cytokine in GCF samples from the EG compared to the CG. The chi‐square test revealed significant heterogeneity among the studies (*I* ^2^ = 84.7%, *p* < 0.001*), leading to the use of a random effects model to combine the primary results. The funnel plot suggested asymmetry and the potential for publication bias. Egger's test (*t* = 6.44, *p* < 0.001*) provided evidence of this bias (Figure 4A,B).

### 3.3 Analysis of Molecular Interactions

**FIGURE 5:**
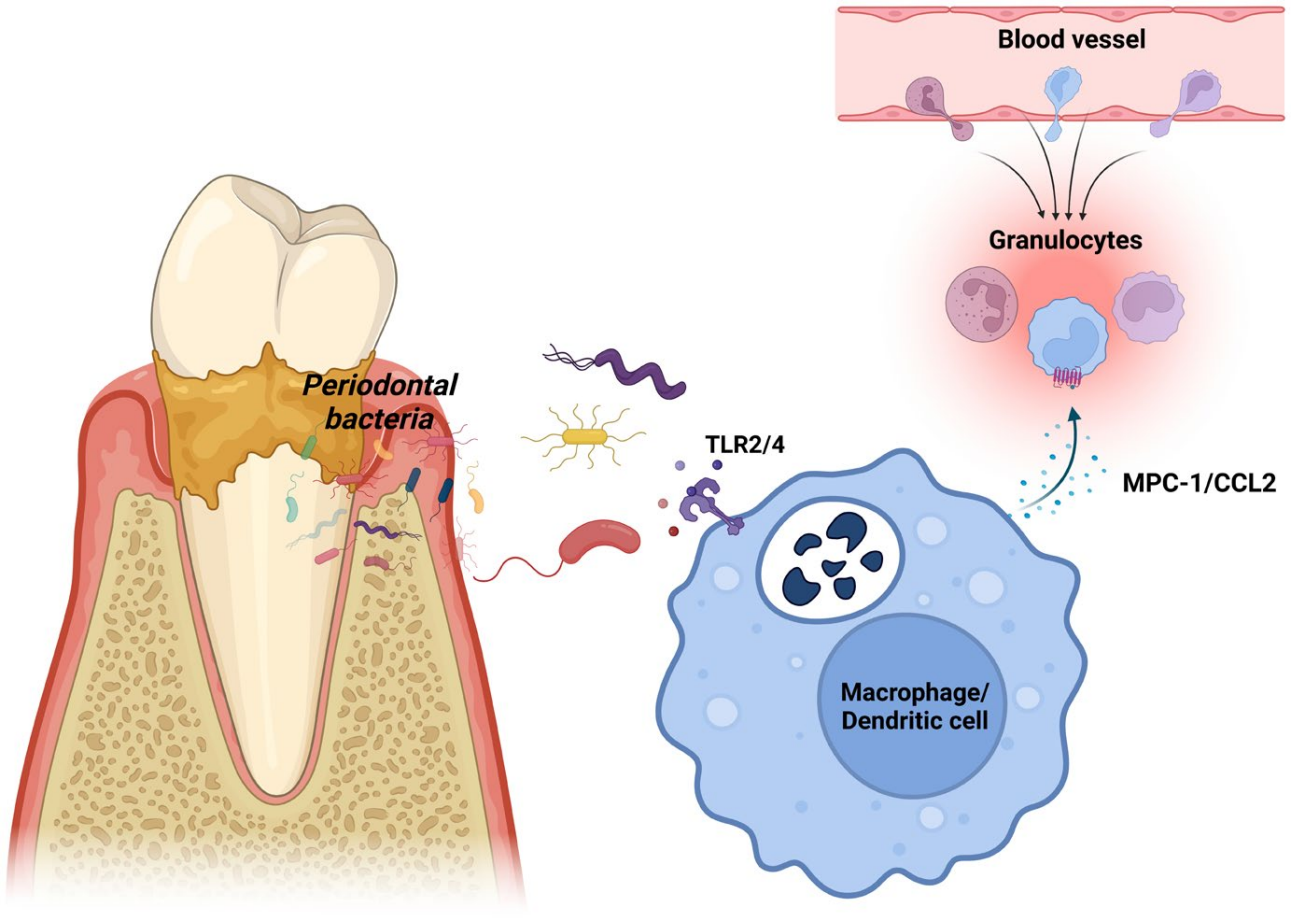
Periodontal bacteria; effect on MCP‐1 expression. Lipopolysaccharide (LPS) from various periodontopathogenic bacteria, including *Porphyromonas gingivalis* , is known to increase MCP‐1 expression in endothelial cells, including macrophages. This suggests that the bacterial component can trigger inflammatory pathways that lead to increased MCP‐1 synthesis.

The analysis in the STRING database showed 50 prominent interactions (Figure 5). According to the evidence published in the literature, we observed that for MCP‐1/CCL‐2, CCR2, CCR5, CCR1, CXCL8, CXCL9, CCL8, CCL5, CCL4L2 and CCL15 there is experimental evidence of their direct interaction with a score of 0.999; followed by CXCL13, PF4, CCL11, CX3CR1, CXCR3, CCL26, CXCR4, CCR3 with 0.998; CXCR2 with 0.997; CXCR1, CCR6, CXCL17 with 0.996; ACKR1 with 0.994; CCRL2 with 0.990; RELA with 0.987; IL6, ACKR2 with 0.985; NFKB1 with 0.982; JUN, CXCL1 with 0.980; CCL20 with 0.979; TNF with 0.978, IL1B with 0.970; CCR7, CXCL10 with 0.965; IL10 with 0.963; IFNG with 0.938; IL1A with 0.934; IL13 with 0.932; CSF2 with 0.931; CSF3 with 0.930; CXCL2 with 0.929; ICAM1 with 0.928; CCR8 with 0.925; IL4 with 0.921; CCR4 with 0.920; SERPINE1 with 0.918; CCL22 with 0.914; and VCAM1 with 0.907.

## 4 Discussion

MCP‐1 plays an important role in the pathogenesis of periodontitis, a chronic oral infection affecting the periodontium [46]. Virulence factors, such as lipopolysaccharide (LPS) from periodontopathogenic species ( *P. gingivalis* ), activate toll‐like receptors on host cells, which induce an increase in MCP‐1 levels by different cell types, such as dendritic cells and macrophages [47]. The release of this chemokine in turn induces the recruitment and activation of immunocytes in foci of inflammation, which is a critical aspect of the immunoinflammatory response associated with this disease [10] (Figure 5). In a local clinical setting, MCP‐1 has been shown to be expressed in gingival tissue and correlates with disease severity in subjects with periodontitis [48]. In addition, this chemokine can be found in biofluids, such as saliva, peri‐implant crevicular fluid (PICF) and GCF [34]. One study demonstrated that MCP‐1 levels were higher in the saliva of individuals with periodontitis compared to subjects with gingivitis and control individuals. For this investigation, ROC curve analysis showed high specificity and sensitivity (100%) for MCP‐1 in discriminating between periodontitis and periodontal health [49]. Despite this, other investigators have reported inverse levels of this chemokine [50, 51], so there is controversy and some inconsistency in the findings reported in the literature. Likewise, in individuals rehabilitated with dental implants, a trend towards elevated levels of MCP‐1 in PICF of dental implants affected by peri‐implantitis compared to healthy implants has been demonstrated [52].

In the first instance, Gürkan et al. demonstrated that the levels of MCP‐1, macrophage migration inhibitory factor (MIF) and activation‐regulated protein expressed and secreted by normal T cells (RANTES) in GCF were increased in subjects with gingivitis compared to gingivally healthy subjects [53]. These findings suggest that MCP‐1 is present in early gingival inflammation.

As the disease progresses, some researchers, such as Gündoĝar et al. [28], Patil et al. [29], Fageeh et al. [30], Zhu et al. [31], Anil et al. [32], Shimada et al. [33], Gupta et al. [34], Thunell et al. [35], Pradeep et al. [36], Pradeep et al. [37], Silva et al. [38], Kurtis et al. [39], Hanioka et al. [40] and Hanazawa et al. [41] found increased levels of MCP‐1 in the GCF of subjects with periodontitis compared to periodontally healthy subjects, as well as positive correlations with periodontal clinical parameters.

Similarly, Emingil et al. [54] found higher levels of MCP‐1 and RANTES in subjects with generalised aggressive periodontitis compared to their control group. In this case, also the levels of these two chemokines were positively correlated with PD and CAL. Similarly, Gunpinar et al. [55] found that all clinical parameters and the total amount of MCP‐1 in GCF were significantly higher in subjects with localised and generalised aggressive periodontitis compared to the control group. This highlights the importance of MCP‐1 with the progression of periodontitis by promoting osteoclastogenesis and promoting bone loss [49]. Therefore, MCP‐1 levels could have important clinical utility as a diagnostic tool to discriminate the different types of periodontal disease.

In addition to locally affecting tissues, MCP‐1 also represents systemic inflammatory processes that could be involved in periodontal tissue degeneration. It has been reported that in response to LPS from *P. gingivalis* , elevated glucose levels increase MCP‐1 expression in human endothelial cells [47]. On the other hand, a literature search revealed that the gingival tissues of diabetic and periodontitis mice showed increased levels of MCP‐1 compared to diabetic mice without periodontitis [56]. Similarly, another study reported a similar trend towards elevated serum MCP‐1 levels in patients with diabetes mellitus and periodontitis compared to diabetic and healthy periodontal individuals [57]. This suggests that metabolic circumstances may worsen periodontal inflammation by inducing increased chemokine synthesis [47]. This explains why people with diabetes have an aggravation of clinical signs and symptoms of periodontitis [58]. These findings highlight the connection between systemic variables and periodontitis, with MCP‐1 acting as a link between metabolic dysregulation and periodontal disease.

**FIGURE 6:**
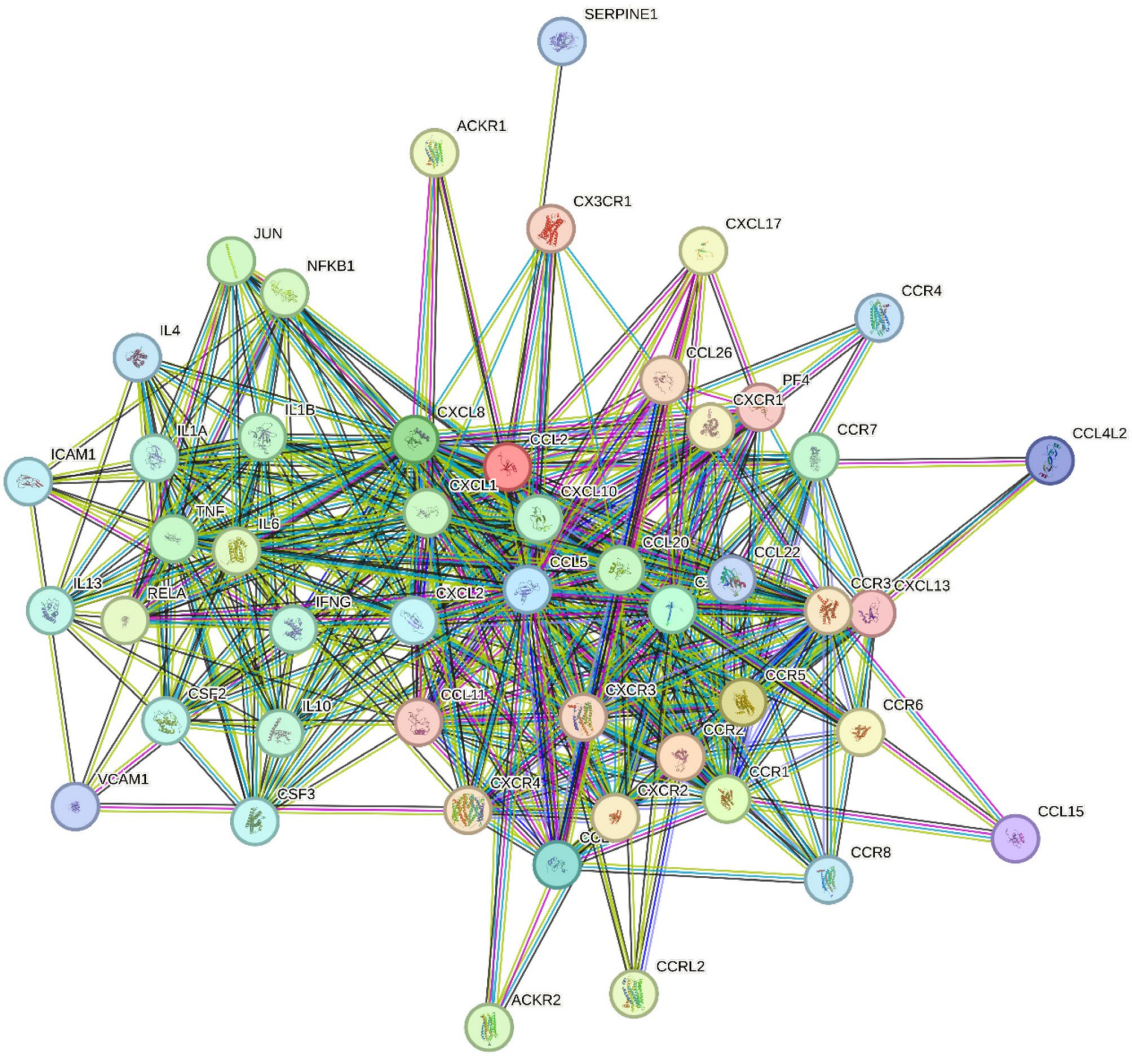
MCP‐1 interactome.

As for the analysis of molecular interactions, we found that MCP‐1 physically interacts with 50 target proteins (Figure 6), of which 22 have been identified in GCF. The receptors CCR1, CCR2, CCR5, CX3CR1, CXCR4, CXCR2 and the ligands CXCL8, CCL5, CCL20, CXCL1, CXCL4 and CXCL10 are potent chemoattractants and regulate leukocyte migration. Studies have shown higher levels of certain chemokines in GCF from individuals with periodontitis than in those without the disease [30, 59, 60, 61, 62, 63, 64, 65, 66, 67]. This increase in leukocytes in periodontal tissues leads to an increase in proinflammatory cytokines. Proinflammatory cytokines, such as TNF‐α, IL‐6, IL‐1β and IL‐1α, contribute to T helper (Th) cell differentiation, increase the expression of receptor nuclear factor kappa B ligand (RANKL), which is involved in bone resorption, and also suppress osteoblastic activity while promoting the production of other proinflammatory cytokines in inflammatory cells, including macrophages, neutrophils, dendritic cells, B cells and T cells. Elevated levels of these cytokines in the GCF of individuals with periodontitis have been confirmed by scientific evidence [68, 69, 70, 71]. The anti‐inflammatory cytokines IL‐4 and IL‐10 increase the production of B and T lymphocytes and also suppress the production of proinflammatory cytokines. Studies indicate reduced levels of these cytokines in GCF from individuals with periodontitis compared to those with a healthy periodontal state [72, 73]. IFN‐γ is a potent activator of macrophages, has antiproliferative effects on transformed cells, and can amplify the antiviral and antitumor responses of type I interferons. Colony‐stimulating factor 2 (CSF2) promotes the growth and differentiation of haematopoietic precursor cells in various lineages. Elevated levels of both IFN‐γ and CSF2 have been observed in the GCF of patients with periodontitis relative to a control group [74, 75]. In addition, ICAM‐1 and VCAM‐1 are involved in leukocyte‐endothelial cell adhesion, and increased levels of these molecules have been observed in the GCF of individuals with periodontitis compared with periodontally healthy individuals [76].

A proinflammatory microenvironment is imminent in periodontitis, due to the increase in different cytokines and chemokines found in gingival tissue, and GCF. It is possible that the expression of these cytokines is positively correlated with the expression of MCP‐1. We consider it important to evaluate this association, which could contribute to the search for biomarkers for pharmacological targeting [77, 78].

In summary, MCP‐1 plays an important role in the inflammatory mechanisms underlying periodontitis. It promotes the recruitment of immune cells that contribute to tissue destruction and has an increased expression in gingival tissues and GCF that correlates with disease severity. Knowing how MCP‐1 functions in periodontitis may help develop new treatment strategies that control inflammation and stop periodontal tissue loss.

## 5 Limitations

This review has provided substantial evidence of the state of the art on the relationship between MCP‐1 levels in GCF and periodontitis; however, we recognise that our study also presents some limitations as shown below: (I) Some studies [30, 34, 40, 41] lacked the necessary statistical information (numerical values) to perform a meta‐analysis; (II) the small sample size of the included studies, which particularly characterises this type of research where the levels of inflammatory mediators in GCF are analysed, may have limited the statistical power of the findings, which can sometimes make it difficult to detect significant differences or to generalise the results to the general population; (III) the inclusion of cross‐sectional studies and randomised trials may introduce variability in study quality and potential bias. Randomised trials generally tend to provide stronger evidence than observational studies, and mixing these designs could complicate the interpretation of overall effects; (IV) studies may not adequately control for potential confounding factors, such as age, gender, oral hygiene, dietary habits, dental conditions or pre‐existing systemic conditions, which could influence GCF cytokine levels independently of periodontal condition; (V) the use of different classifications to determine the patient's periodontal status, as well as the different techniques and immunoassay protocols of the different studies, could lead to variability in the measurement of MCP‐1 concentrations, which would affect the comparability of the results.

## 6 Conclusions

GCF MCP‐1 levels are elevated in periodontitis compared to healthy controls, so they could be used in the future in the clinic as diagnostic tools. Likewise, the information presented here generates new knowledge about the levels of a potential biomarker in subjects with periodontitis.

## Author Contributions

**Mario Alberto Alarcón‐Sánchez:** conceptualization (equal), data curation (equal), formal analysis (equal), investigation (equal), methodology (equal), project administration (equal), resources (equal), software (equal), supervision (equal), validation (equal), visualization (equal), writing – original draft (equal), writing – review and editing (equal). **Ruth Rodríguez‐Montaño:** conceptualization (equal), formal analysis (equal), investigation (equal), project administration (equal), resources (equal), supervision (equal), validation (equal), visualization (equal), writing – original draft (equal), writing – review and editing (equal). **Sarah Monserrat Lomelí‐Martínez:** formal analysis (equal), visualization (equal), writing – original draft (equal). **Artak Heboyan:** project administration (equal), supervision (equal), validation (equal), visualization (equal), writing – review and editing (equal).

## Ethics Statement

The authors have nothing to report.

## Consent

The authors have nothing to report.

## Conflicts of Interest

The authors declare no conflicts of interest.
